# Weight Loss and Engagement Among Active Users of a Dietitian-Led Digital Nutrition Platform: Retrospective Cohort Study

**DOI:** 10.2196/94732

**Published:** 2026-09-15

**Authors:** Frederick G B Goddard, Jessica Garay, Francisco Teixeira Neves, Alexandra Ligeti, Liudmila Zhaunova, Manuela Abreu

**Affiliations:** 1 Healthium - Healthcare Software Solutions, S.A., Rua Andrade Corvo, n.º 242, First Floor, Room 106, Braga, 4700-204 Maximinos, Portugal, +351 935 455 758; 2 Department of Nutrition and Food Studies, Syracuse University, Syracuse, NY, United States

**Keywords:** obesity, digital, nutrition, dietitian, weight

## Abstract

**Background:**

Approximately 2.5 billion adults worldwide were living with overweight or obesity as of 2022, highlighting the need for comprehensive weight management solutions. Digital nutrition platforms have emerged as an accessible solution that can be implemented at scale. Nutrium Care is an employer-sponsored program delivered via the Nutrium platform, facilitating dietitian-led nutrition counseling through consultations and in-app communication, while also enabling users to track food intake, water consumption, and physical activity.

**Objective:**

This study set out to characterize longitudinal weight changes among active users of the Nutrium Care program and estimate associations between program engagement and weight loss.

**Methods:**

This retrospective cohort study analyzed deidentified data from adult users with overweight or obesity enrolled in the Nutrium Care program. Users were included if they were taking action to manage their weight and had at least 3 months of follow-up data available; these users were referred to as active users. The primary outcome was weight loss after 3 and 6 months, estimated using piecewise linear mixed-effects models. To estimate the association between engagement and weight loss, more- and less-engaged users were matched on sociodemographic characteristics. Engagement was defined by the number of dietitian appointments, days of active platform use, and logging of food, water intake, and physical exercise.

**Results:**

The study included 10,288 active users, predominantly female (n=6887, 66.9%) and aged 18 to 49 years (n=9741, 94.7%), who contributed a median of 6 (IQR 4‐9) months of data to the cohort. In the first 3 months, active users lost 2.0 kg (2.4% of baseline body weight; 95% CI 1.9-2.0 kg) on average, increasing to 2.3 kg (2.8%; 95% CI 2.2-2.4 kg) by 6 months. Higher program engagement was associated with more weight loss, with the strongest association estimated for at least 1 dietitian appointment every 2 months, corresponding to 0.8 kg (95% CI 0.7-1.0 kg) additional weight loss after 3 months.

**Conclusions:**

This retrospective cohort study addresses a critical research gap in hybrid nutrition interventions integrating professional dietitian support with an employer-sponsored digital nutrition program. Active users achieved weight loss, and regular contact with a dietitian was the strongest engagement factor associated with improved outcomes, supporting the potential role of professional support in digital weight management programs.

## Introduction

The global prevalence of adult overweight and obesity has risen in recent decades and now represents one of the most significant public health challenges worldwide [1]. As of 2022, a total of 2.5 billion adults were living with overweight or obesity globally [2]. Obesity substantially increases the risk of chronic diseases, including type 2 diabetes, cardiovascular disease, and certain cancers [3], and contributes to an estimated 4 million deaths annually [4]. The associated economic burden was estimated to reach US $2.5 trillion in 2025 [5,6], highlighting the urgent need for scalable and sustainable solutions to weight management.

Multiple strategies have been used to support weight loss, including bariatric surgery, prescription medication (ie, appetite suppressants), and lifestyle interventions [7]. Recently, pharmacotherapy, particularly glucagon-like peptide-1 (GLP-1) receptor agonists, has gained significant attention due to its demonstrated effectiveness in reducing body weight [8]. However, long-term use of these drugs has not yet been well studied, and there are potential concerns related to loss of muscle mass and other health effects that warrant ongoing monitoring [9]. Lifestyle interventions that combine dietary modification with physical activity remain a cornerstone of obesity management and are particularly effective when delivered with guidance from health care professionals, such as registered dietitians (RDs) [10,11].

Despite their effectiveness, lifestyle interventions delivered through in-person counseling are difficult to scale to large populations. The emergence of digital health and nutrition platforms offers an alternative scalable approach to supporting weight management. These platforms typically combine individualized nutrition counseling with tools for dietary self-monitoring, educational resources, and behavioral prompts designed to encourage sustained lifestyle change. Although these platforms vary in their specific content and features, initial evidence suggests that mobile and web-based health technologies can effectively support improved diet quality and weight loss among adults with overweight or obesity [12-14].

However, digital nutrition platforms differ substantially in their design, level of professional involvement, and behavioral support mechanisms [15]. Evaluations of digital nutrition platforms consistently identify motivation, education, and support features as core user needs [16,17]. User engagement with specific digital intervention features is an important component of achieving weight loss outcomes. Use of self-regulation techniques has been identified as a predictor of successful weight loss among users of digital nutrition platforms [18,19]. A recent meta-analysis of digital food-focused interventions found positive effects on food consumption, particularly if the intervention used prompts or cues [20]. Positive cues that reinforce healthful behaviors can help to combat the “food noise” (persistent thoughts about food) many individuals report as a barrier to successful weight management [21]. Indeed, many GLP-1 users attribute the reduction in “food noise” to their weight loss [22]. However, the specific types and intensity of engagement associated with optimal weight outcomes remain unclear, particularly in dietitian-led platforms.

Inclusion of tailored feedback or individualized counseling has been demonstrated to enhance participant success in digital interventions focused on changing eating behavior [23-25]. An evaluation of a web platform in Brazil concluded that use of the platform in combination with coaching from a dietitian led to greater weight loss and significant changes in eating behavior (more fruits and vegetables, less ultraprocessed food) compared with users who only accessed the platform on their own [26]. Although limited in scope, these results suggest a high level of effectiveness when digital nutrition platforms include access to a dietitian. While the overall utility of digital nutrition platforms is generally positive, the heterogeneity in features, including the level of dietitian support, underscores the need for platform-specific evaluations. This allows for identification of individual components that best enhance weight management outcomes.

**Figure 1.**
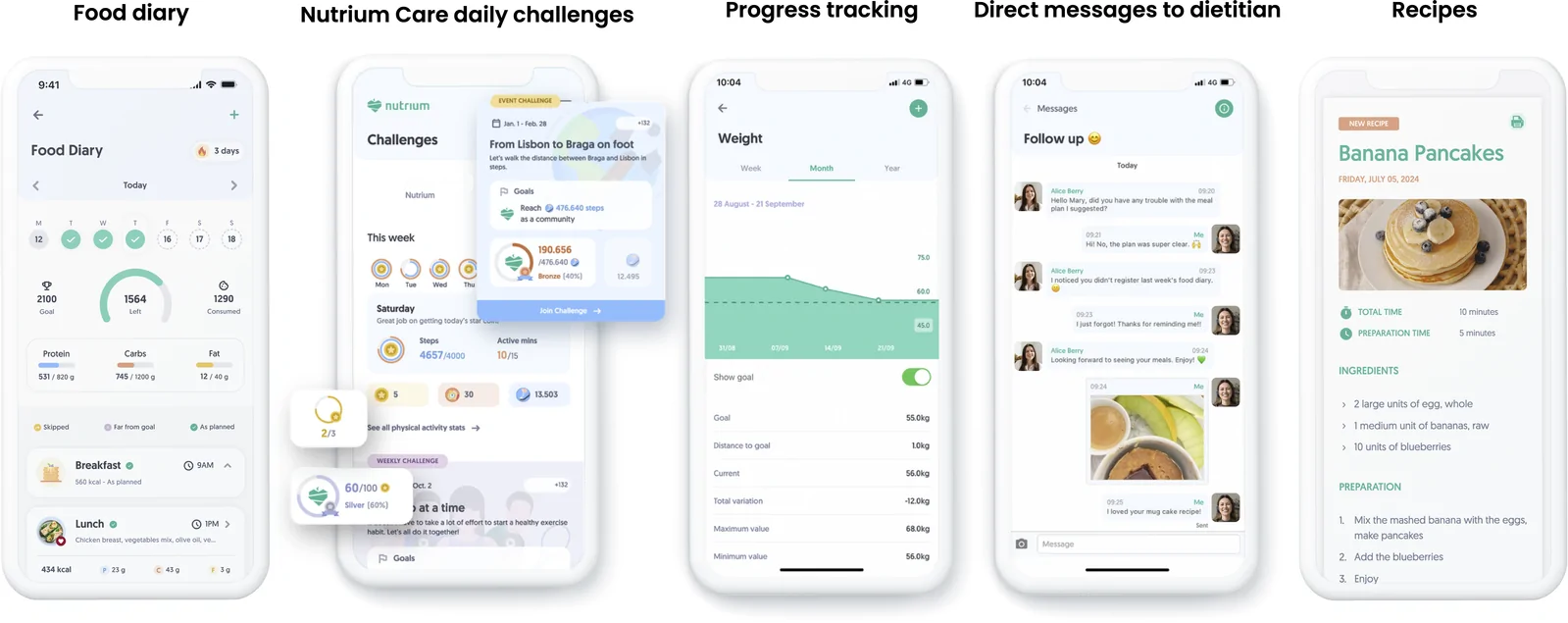
Examples of the in-app user interface.

Nutrium is a digital health platform designed to facilitate hybrid nutrition interventions through integrated asynchronous and synchronous communication between users and RDs. Unlike automated or self-guided nutrition applications, which rely on algorithmic feedback [27], this model centers on practitioner-led care. The platform integrates scheduled teleconsultations with continuous in-app messaging, allowing RDs to develop personalized nutritional interventions, monitor real-time dietary intake, and provide iterative feedback throughout the care continuum. By digitizing the dietitian user interface while maintaining professional oversight (Figure 1), the Nutrium platform aligns with behavioral frameworks that prioritize engagement and therapeutic adherence as primary drivers of long-term health outcomes, such as weight management [28].

Nutrium Care is one delivery model for the Nutrium platform, implemented via employer-sponsored health and wellness programs. Through this model, employees gain access to professional dietetic consultations and sustained nutritional support through the Nutrium platform. This study aimed to provide the first evaluation of the Nutrium Care delivery model by quantifying longitudinal weight loss outcomes among program users and assessing the relationship between weight loss and user engagement with the program. Specifically, the study aimed to (1) characterize absolute weight loss among active Nutrium Care users after 3 and 6 months; (2) describe the proportion of active users achieving weight loss thresholds of 3%, 5%, and 10% of baseline body weight in the action phase; and (3) estimate the association between user engagement with the program and weight loss across multiple engagement metrics, including dietitian appointments, days of active platform use, and logging of food, water intake, and physical exercise.

## Methods

### Study Design

This study was a retrospective analysis of secondary observational data from users of the Nutrium Care program, specifically examining user weight outcomes and engagement with the app. The study was completed in 2 parts. First, we examined longitudinal weight data to estimate weight outcomes 3 and 6 months after users joined the Nutrium Care program. Second, we quantified the difference in weight outcomes at 3 months between more and less engaged users.

This evaluation considered users’ readiness to engage with behavior change. The transtheoretical model (TTM) conceptualizes behavior change across 5 core stages: precontemplation, contemplation, preparation, action, and maintenance [29]. The action phase reflects individuals who have moved beyond intention into active, committed behavior change. Here, users who attended at least 2 dietitian appointments, with the second occurring within 6 months of baseline, were classified as action phase users [30,31]. This study therefore restricted its evaluation to users whose platform engagement was consistent with active behavior change, rather than incidental or exploratory use, by considering only users in the action phase. This restriction was applied during revision to ground the analysis in the TTM, rather than being prespecified. For the remainder of this manuscript, we refer to users in the action phase as active users.

To estimate differences in weight outcomes by engagement, we compared more and less engaged users matched on sociodemographic characteristics. For each engagement metric, we had 2 groups: one that included users who met the threshold for engagement (more engaged users) and one that included users who did not (less engaged users). Engagement metrics and their thresholds were appointments with dietitians (≥1 appointment at least every 2 months), days of active use (DAU) of the platform (≥10 DAU per month), and logging of food, water intake, and physical exercise (≥1 time per week on average). As no established thresholds exist across the included engagement metrics for digital nutrition platforms, the thresholds applied were exploratory and defined a priori by the research team.

### Study Population

The study population included Nutrium Care users aged 18 to 65 years with overweight (BMI 25‐29.9), obesity (BMI 30‐39.9), or morbid obesity (BMI ≥40) at baseline. Baseline was defined as the date of the first available weight measurement. Users were excluded if they did not have a valid baseline weight, which we defined as a weight measured within 30 days of their first appointment with a dietitian, had missing data on age or sex, reported using weight loss medications (ie, GLP-1 agonists), or reported a pregnancy. Implausible weight (<30 kg or >300 kg) and BMI (>60) data were filtered out before applying the inclusion and exclusion criteria [32]. Users needed to be in the action phase and have at least 3 months of follow-up data to be included in the primary analysis. Results for an unrestricted sample without action phase and follow-up restrictions are presented alongside the primary analysis.

### Data

The data consisted solely of information that was originally generated during ordinary, routine use of the Nutrium Care program. Participants were not prompted to enter data for research purposes. Instead, all information was submitted voluntarily in the normal course of platform operation and service delivery. The data were entered by dietitians as well as self-reported by users. Data extracted from the Nutrium database included sociodemographic variables (age and sex), anthropometrics (weight and height), and engagement with the Nutrium application (including the number of dietitian appointments, exercise, food and water intake logging, DAU, and dietary goals). Users recorded weight by either entering a value manually in the application, reporting it to their dietitian during a consultation for entry into the user’s record, or synchronizing a connected smart scale with user permission, which transmitted the weight automatically each time users weighed themselves. All datasets were deidentified prior to analysis. The research team had access only to pseudonymized or anonymized records containing no direct identifiers, and no attempt was made to re-identify individuals.

### Outcomes

The primary outcome was weight loss in kg and as a percentage of baseline body weight at 3 and 6 months. Secondary outcomes included the proportion of users achieving any weight loss as well as weight loss thresholds of 3%, 5%, and 10% of baseline body weight at each time point.

### Statistical Analysis

#### Weight Outcomes at 3 and 6 Months

To estimate average weight change at 3 and 6 months compared with baseline, we fit piecewise linear mixed-effects models. Time was represented with piecewise linear terms with break points at 3 and 6 months, allowing for different rates of weight change over time. We chose these time points based on the hypothesis that dietary behavior changes would be most intensive initially (0 to <3 months), continue at a slower rate (3 to <6 months), then stabilize (≥6 months). The model included random effects for the intercept and the overall time slope to account for between-user variation in baseline weight and weight change over time. Weight loss at 3 and 6 months was estimated by computing linear combinations of the fixed effects from the piecewise linear terms. We stratified the analysis by sex, age group, and baseline BMI category.

#### Weight Loss Thresholds

For the proportion of active users who achieved weight loss thresholds at each time point, we identified the weight measurement closest to each respective time point and compared it with the user’s baseline weight. Users with no measurements within 30 days of a given time point were excluded from the primary threshold analysis for that time point. All users recorded a weight at or beyond 3 months, so exclusions at the 3-month time point reflect measurement timing rather than absent outcome data. At 6 months, approximately half the cohort had no recorded weight at or beyond that point. We therefore report the 6-month thresholds as a range, with the measured-only proportion as the upper bound and a lower bound assuming that every user without a measurement near the time point did not reach the threshold, using the full analyzed cohort as the denominator. Additionally, as a sensitivity analysis, we repeated the threshold analysis using the last nonbaseline weight observation carried forward for excluded users. Users with no eligible prior measurement remained excluded.

#### Weight Outcomes by Engagement

To quantify the difference in weight change by engagement status, we first created a matched sample for each engagement metric. We used coarsened exact matching (CEM) [33] to balance groups of more engaged users with less engaged users on the following criteria: age group (18-29, 30-49, or 50-65 years), sex, country of residence, and BMI at first appointment (25‐29.9, 30‐39.9, or ≥40). Then we fit weighted piecewise linear mixed-effects models to the matched datasets. The model included an interaction term between engagement status and time for each of the 3 time intervals (0 to <3, 3 to <6, and ≥6 months). Because all included users had measurements until at least the 3-month time point, we estimated differential rates of weight change between more and less engaged users at this time point only. The models were weighted by applying the CEM weights to account for any residual imbalance between engagement groups after matching. Separate models were fit for each included engagement metric.

All analyses were conducted independently by the lead author. A coauthor reviewed the data processing pipeline against the inclusion and exclusion criteria for data quality issues. All analyses were completed in R (version 4.4.0; R Foundation for Statistical Computing) [34].

### Ethical Considerations

The study received an exempt determination from the Syracuse University Institutional Review Board (protocol number 26‐018). During account registration, users must review and consent to Nutrium’s privacy policy before creating an account or providing any personal data. The policy explained how personal data may be processed in connection with platform use and informed users that data may be deidentified and aggregated for research and analytical purposes. For this study, only deidentified data were used, and no directly identifiable personal data were accessed by the research team. Users did not receive any compensation for participation in this study.

## Results

### Study Sample

**Figure 2.**
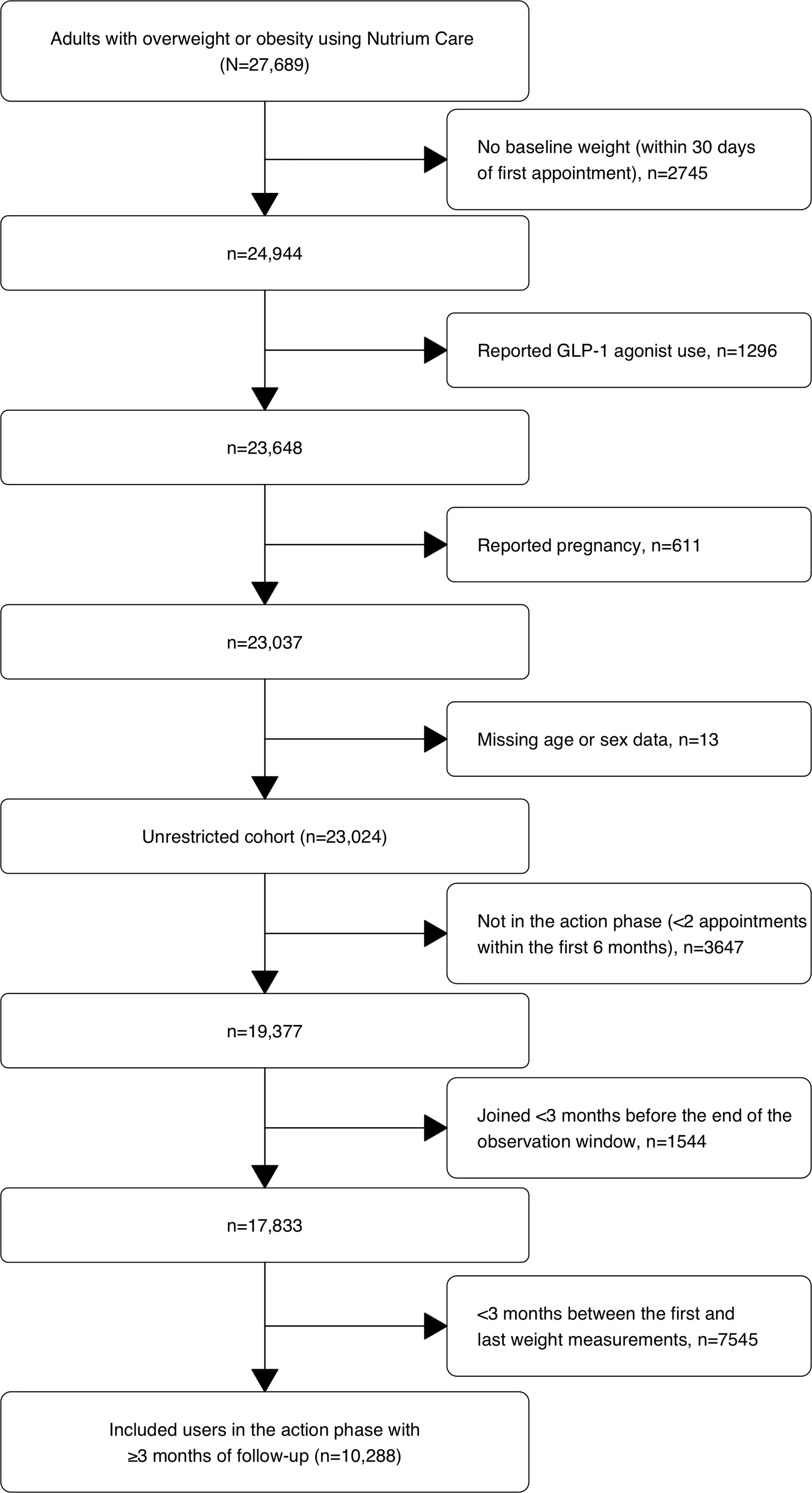
Study population.

The population considered for this study included 27,689 adults with overweight or obesity using Nutrium Care (Figure 2). The unrestricted sample, after excluding users with no baseline weight measurement (2745/27,689, 9.9%), reported GLP-1 agonist use (1296/24,944, 5.2%), reported pregnancy (611/23,648, 2.6%), or missing data on age or sex (13/23,037, 0.1%), included 23,024 users. The sample analyzed for the primary action phase population included 44.7% (10,288/23,024) of eligible users from the unrestricted population, after excluding 3647 (15.8%) users who were not in the action phase (<2 appointments in the first 6 months), 1544 (6.7%) users who joined within 3 months of the end of the observation window and had no opportunity to accrue 3 months of follow-up, and 7545 (32.8%) users who had fewer than 3 months between their first and last recorded data points.

Two-thirds of included users were female (6887/10,288, 66.9%), and most were aged 18 to 29 years (4584/10,288, 44.6%) and 30 to 49 years (5157/10,288, 50.1%; Table 1). The study included 61.2% (6293/10,288) of users with overweight, 34.3% (3531/10,288) of users with obesity, and 4.5% (464/10,288) of users with morbid obesity at baseline. Most users in this sample joined the platform in 2024 or 2025, contributing a median of 6 (IQR 4‐9) months of data and 5 weight measurements to this study (Table 1). Follow-up duration was the number of months between a user’s first and last recorded weight measurements. During the first 6 months, users had a median of 3 (IQR 2-4) dietitian appointments and 25 (IQR 14-44) days of platform app use and logged food, water intake, and exercise a median of 6 (IQR 2-25), 3 (IQR 1-14), and 3 (IQR 0-21) times, respectively (Table 1).

**Table 1.** Study population sociodemographic characteristics, data contributed, and engagement metrics in the first 6 months (N=10,288).

	Values
Sociodemographic characteristics	
Sex, n (%)	
Female	6887 (66.9)
Male	3401 (33.1)
Age (y), n (%)	
18‐29	4584 (44.6)
30‐49	5157 (50.1)
50‐65	547 (5.3)
BMI category, n (%)	
Overweight	6293 (61.2)
Obesity	3531 (34.3)
Morbid obesity	464 (4.5)
Data contributed	
Platform registration year, n (%)	
<2022	8 (0.1)
2022	175 (1.7)
2023	293 (2.8)
2024	4070 (39.6)
2025	5742 (55.8)
Months in the cohort, median (IQR)	6 (4-9)
Users with a minimum of 6 months of follow-up data, n (%)	5343 (51.9)
Weight measurements contributed, median (IQR)	5 (3-7)
Engagement in the first 6 months, median (IQR)	
Number of appointments	3 (2-4)
Days of active use	25 (14‐44)
Food diary logs	6 (2-25)
Water intake logs	3 (1-14)
Exercise logs	3 (0‐21)

### Weight Outcomes at 3 and 6 Months

**Figure 3.**
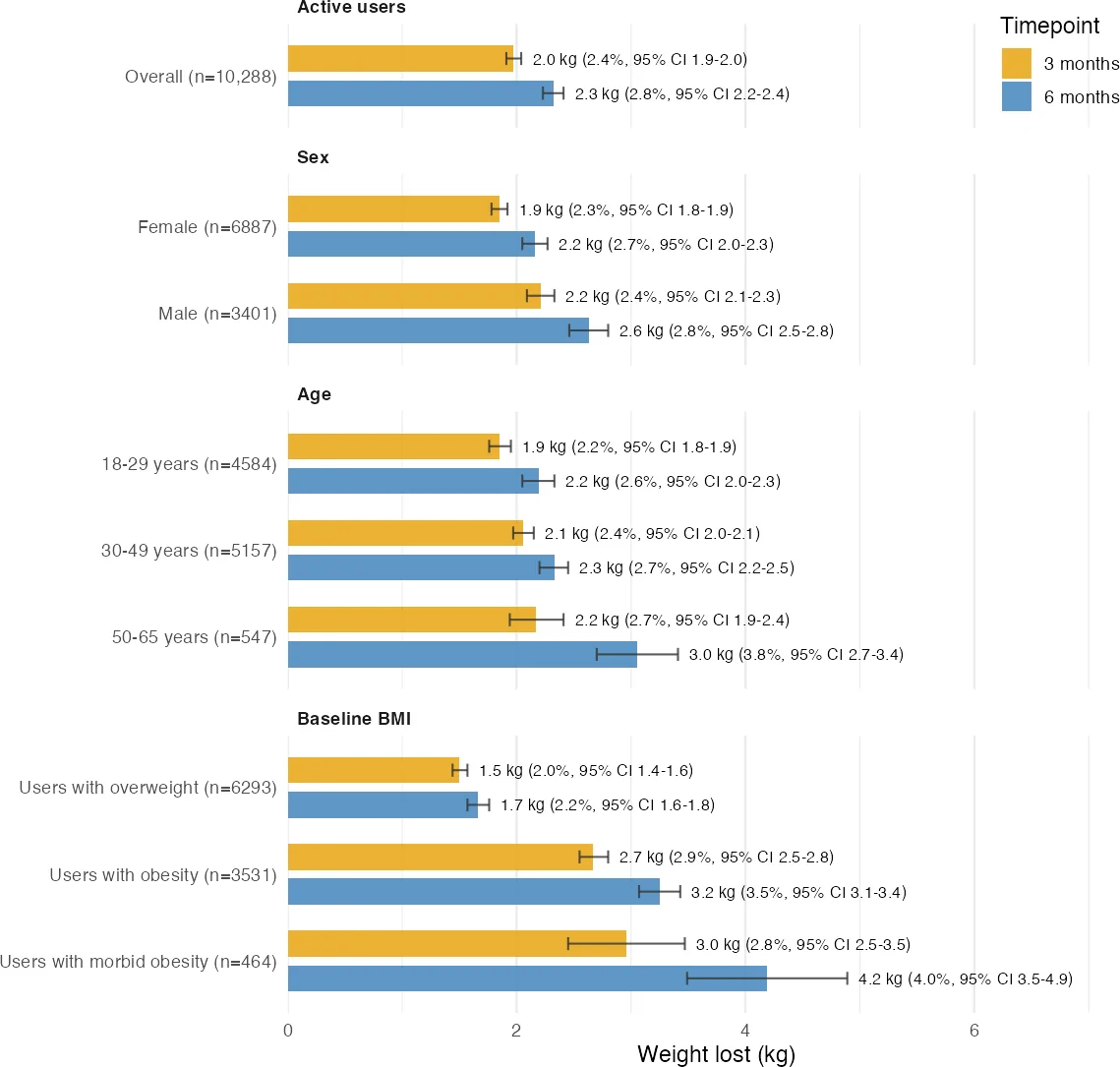
Absolute weight loss at 3 and 6 months among active users (*P*<.001 for all estimates). A total of 22,043 weight measurements beyond baseline were recorded in the 0-3 month period and 18,641 in the 3-6 month period, contributed by 7941 and 8727 users, respectively.

Three months after joining Nutrium Care, active users lost 2.0 kg on average (2.4% of their body weight; 95% CI 1.9‐2.0 kg; Figure 3). By 6 months, average weight loss increased to 2.3 kg (2.8%; 95% CI 2.2‐2.4 kg). Weight loss was slightly more pronounced among male users and among users aged 50 or older, although this sample of users in this age group was small (n=547, 5.3%), and varied substantially by baseline BMI at both 3 and 6 months. At 6 months, users with overweight lost 1.7 kg on average (2.2%; 95% CI 1.6‐1.8 kg), whereas users with obesity averaged 3.2 kg of weight loss (3.5%; 95% CI 3.1‐3.4 kg) and users with morbid obesity averaged 4.2 kg (4%; 95% CI 3.5‐4.9 kg). In the unrestricted sample, mean weight loss was 2.2 kg (2.6%; 95% CI 2.2‐2.3 kg) at 3 months and 2.4 kg (2.9%; 95% CI 2.4‐2.5 kg) at 6 months (Figure S1 in Multimedia Appendix 1).

### Weight Loss Thresholds

**Figure 4.**
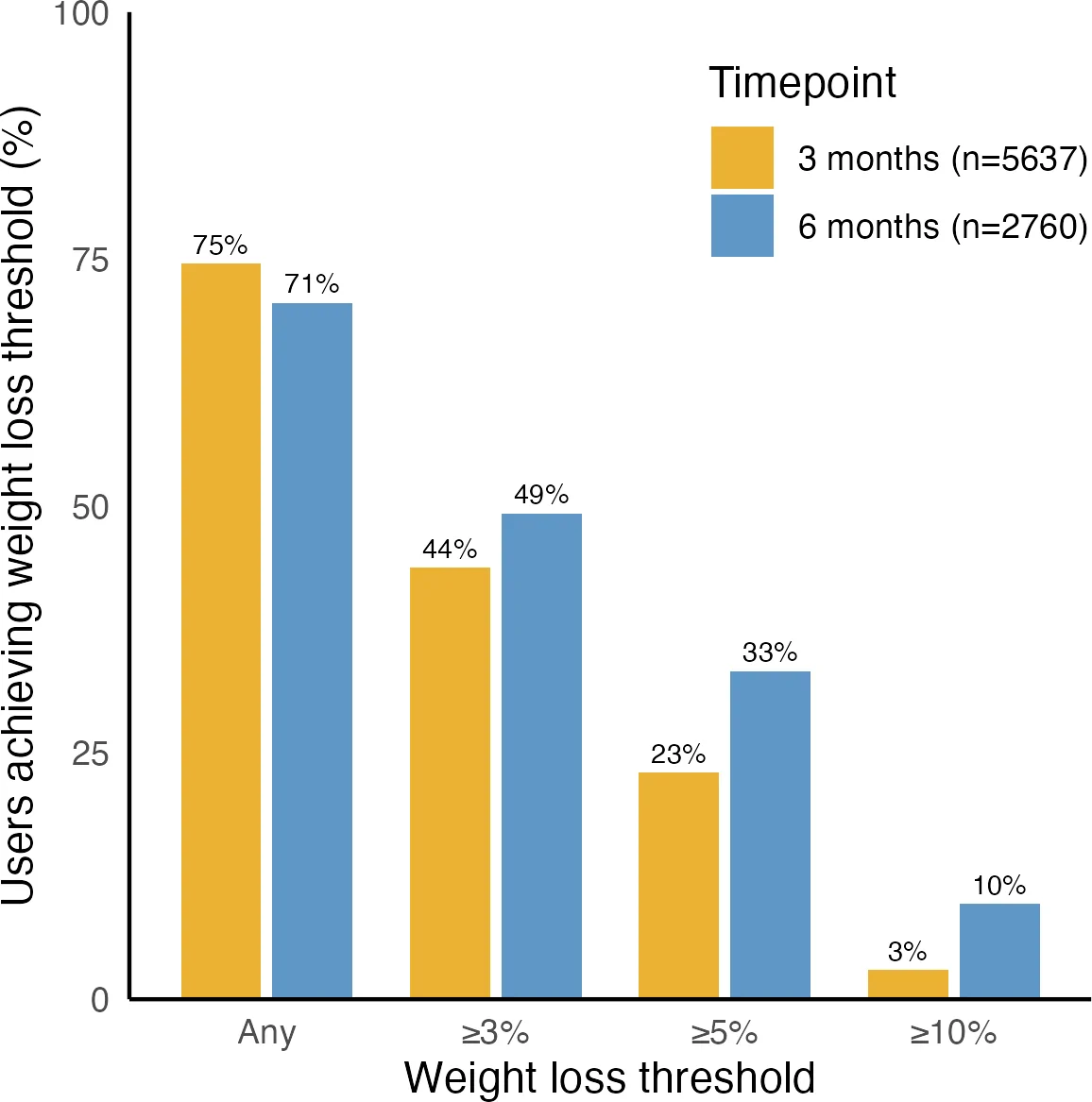
Threshold weight outcomes at 3 and 6 months among active users. Users without a weight measurement within 30 days of the time point were excluded.

At 3 months, weight was available within 30 days of the time point for 54.8% (5637/10,288) of active users. Among these, most (4200/5637, 74.5%) had lower weight compared with baseline (Figure 4). Nearly half of these active users (2464/5637, 43.7%) achieved at least 3% weight loss, nearly one quarter (1293/5637, 23%) achieved at least 5%, and a smaller proportion (166/5637, 2.9%) at least 10% weight loss at this time point. All users in the action phase cohort recorded a weight at or beyond 3 months, so users excluded at this time point had recorded a weight outside the 30-day window rather than having stopped weighing in.

At 6 months, weight was available within 30 days of the time point for 26.8% (2760/10,288) of active users. Among these, a similar proportion of active users (1947/2760, 70.5%) weighed below their baseline weight, almost half (1358/2760, 49.2%) achieved at least 3% weight loss, one third (917/2760, 33.2%) achieved at least 5%, and one in 10 (266/2760, 10%) achieved at least 10% weight loss. Because 52% (5343/10,288) of active users accrued 6 months of follow-up, these proportions are restricted to active users who continued recording weights and are likely to be optimistic. Assuming instead that every active user without a weight near the time point did not reach the threshold gives lower bounds of 18.9% (1947/10,288) for any weight loss, 13.2% (1358/10,288) for 3% weight loss, 8.9% (917/10,288) for 5% weight loss, and 2.6% (266/10,288) for 10% weight loss. In the unrestricted sample, 48.5% (1377/2840) of users lost at least 3% of baseline weight at 6 months, 33% (929/2840) lost at least 5% and 9.4% (268/2840) lost at least 10% (Figure S2 in Multimedia Appendix 1). A sensitivity analysis that carried the last observed weight measurement forward among active users gave intermediate estimates between the primary estimates and lower-bound estimates presented here (Figure S3 in Multimedia Appendix 1).

### Weight Outcomes by Engagement

Active users who met the engagement criteria had greater weight loss at 3 months compared with matched users who did not meet these criteria (Table 2). Among the engagement metrics considered, the largest differences were observed for users with more dietitian appointments. Users with at least 1 appointment every 2 months (bimonthly appointments) experienced 0.8 kg (95% CI 0.7‐1.0) greater weight loss at 3 months compared with matched users without bimonthly appointments.

**Table 2.** Difference in weight loss at 3 months by engagement metric (N=10,288).

Engagement metric	Participants, n (%)	Weight loss difference (kg; 95% CI)^a^	
Bimonthly appointment	7392 (71.9)	0.8 (0.7‐1.0)	
≥10 days of active use per month	2344 (22.8)	0.7 (0.6‐0.9)	
Weekly food logging	3126 (30.4)	0.4 (0.3‐0.6)	
Weekly water intake logging	2207 (21.5)	0.4 (0.2‐0.5)	
Weekly exercise logging	2799 (27.2)	0.3 (0.2‐0.5)	

a All estimates, *P*<.001.

Users with at least 10 DAU per month experienced 0.7 kg (95% CI 0.6‐0.9) more weight loss. Logging behaviors showed less pronounced associations with weight loss at 3 months. Weekly food diary loggers experienced 0.4 kg (95% CI 0.3‐0.6) additional weight loss, weekly water intake loggers experienced 0.4 kg (95% CI 0.2‐0.5), and weekly physical exercise loggers experienced 0.3 kg (95% CI 0.2‐0.5) additional weight loss compared with matched users who logged less frequently. Differences in weight loss by engagement metric were moderately larger in the cohort before the action phase restriction, with similar ordering across metrics (Table S1 in Multimedia Appendix 1).

## Discussion

This study evaluated real-world longitudinal data among more than 10,000 users with overweight and obesity enrolled in the Nutrium Care program who progressed to the action phase, reflecting active commitment to achieving their weight goals. Active users lost, on average, 2.0 kg and 2.3 kg after 3 and 6 months, respectively. Among active users with a weight recorded near the 3-month time point, 74.5% (4200/5637) weighed less than at baseline, 43.7% (2464/5637) lost at least 3% of their baseline weight, 22.9% (1293/5637) lost at least 5% of their baseline weight, and 2.9% (166/5637) lost at least 10% of their baseline weight. Greater program engagement was associated with more weight loss across the included engagement metrics, with bimonthly dietitian appointments showing the strongest association with improved weight loss outcomes.

The findings reported in this study are consistent with the growing evidence supporting digital nutrition platforms [35]. The weight loss observed is comparable to outcomes reported for digital interventions and is consistent with losses reported in meta-analyses of smartphone-based apps [36]. Among active users with a weight recorded near the 3-month time point, 23% (1293/5637) reached at least 5% weight loss. The 5% threshold is the benchmark most commonly used to define clinically significant weight loss [37]. Some evidence indicates benefits for cardiovascular risk factors at more modest losses [38], although this is not the established standard. While current guidelines indicate that individuals with obesity will see the biggest health benefits when weight loss exceeds 10% of body weight, most experts agree that these amounts are more likely to result from surgical or pharmaceutical interventions rather than lifestyle changes [39-41].

Behavior change and lifestyle modification have been identified as integral components of successful weight management, including external support from health care providers such as dietitians [42]. Incorporation of techniques that encourage self-regulation, such as tracking hunger levels, mood, and other common cues associated with eating habits, can support increased awareness of an individual’s environmental triggers that either facilitate or derail weight loss attempts [43]. However, simple tracking activities such as recording weight have been shown to be effective, particularly when paired with goal setting within a digital platform [44,45]. In this context, the engagement metrics assessed in the present study may reflect not only platform use but also behaviors associated with sustained participation in weight management.

The association between engagement and weight loss further contextualizes these results within the broader literature [46,47]. Active users attending bimonthly dietitian appointments lost 0.8 kg more than matched users without this level of contact, suggesting a relationship between professional interaction and weight loss [48,49]. These findings are consistent with evidence suggesting that digital interventions that include a component of actual human contact are more effective than fully automated programs [35] and that nutrition coaching, including medical nutrition therapy, delivered via telehealth can be as effective as traditional clinical nutrition care [11,48]. Within the TTM framework, behavioral processes of change such as helping relationships and reinforcement management are most prominent during the action phase, so forms of professional support that provide reinforcement may be particularly relevant for users working to sustain new routines [29]. Dietitian contact showed a stronger association with weight loss than self-directed platform engagement, although the design cannot establish why. However, in the absence of established benchmarks, the thresholds applied to engagement metrics were exploratory, so the estimated differences between more and less engaged users should be limited to hypothesis-generating interpretation. Further, statistical significance does not indicate clinical importance. The differences observed here, ranging from 0.3 kg to 0.8 kg at 3 months, may not be clinically meaningful for individual users.

The findings from this study should be interpreted considering its limitations. First, we relied on secondary observational data with no control group or comparison with other digital nutrition platforms. Therefore, we cannot establish causality in the weight loss results documented here, nor can we directly compare with the effectiveness of other weight loss solutions. Second, both criteria defining the action phase cohort depend on behavior after baseline. Users met the action phase definition only by attending a second dietitian appointment within 6 months, and follow-up was measured from a user’s first to last weight measurement, excluding those who stopped generating data before 3 months. Together, these criteria excluded 48.6% (11,192/23,024) of eligible users, compared with 6.7% (1544/23,024) excluded for joining too late to accrue 3 months of data. The cohort therefore represents users who remained active, and estimates should not be extrapolated to all enrolled users or to employer-sponsored digital nutrition programs more broadly. The action phase criterion is also conceptually adjacent to our strongest engagement exposure, since both are defined by continued dietitian attendance, so the engagement comparison is made within a group already selected on a related behavior. Estimates in the cohort without the action phase restriction were not smaller than our primary estimates, which offers no indication that the restriction inflated our findings, although that cohort includes many users whose values at 3 and 6 months are extrapolated from the fitted model rather than observed, so it cannot serve as a reliable counterfactual. Third, weight measurements were not recorded on a fixed schedule, so the threshold analyses depend on data availability near each time point. At 3 months, this reflects measurement timing rather than absent data, since all users recorded a weight at or beyond 3 months and those excluded had weighed in outside the 30-day window. At 6 months, approximately half the cohort had no recorded weight at or beyond that point. Users who stall or regain weight are more likely to stop recording weights, so the 6-month proportions based on measured weights alone are likely to overstate achievement of each threshold. We therefore report the lower bound of these proportions. Fourth, we were restricted in the length of follow-up. We addressed this by examining 2 time points, 3 and 6 months. However, further evidence on long-term weight loss maintenance, including after discontinuation of platform use, is needed. Fifth, this study was limited to weight loss as an outcome and does not include data on user experiences during the care they received and its impact on well-being. Sixth, the engagement analysis could not control for all possible confounders and potential reverse causality. For example, we did not have data on comorbidities experienced by users. Further, users experiencing difficulty with weight loss may be more engaged with the program, potentially biasing our estimate toward the null. Finally, we relied on data entered both by dietitians and self-reported by users, which may be subject to measurement error and reporting bias.

Our findings support the use of digital nutrition platforms as a meaningful tool to aid in weight management among users motivated and in the action phase of behavior change. Given the reality that primary care providers have limited time to incorporate behavioral change techniques during routine appointments, encouraging users with overweight or obesity to engage with a dietitian via a digital platform will increase access to the services and support needed. Having virtual access to a dietitian increases equity in care and reduces the logistical burden users experience associated with transportation and scheduling challenges that often occur with in-person appointments. Indeed, nutrition interventions using telehealth modalities are more cost-effective overall, with digital (mobile health) rated as the most cost-effective [50]. As employers (and health insurance companies) expand health benefits to include nutrition services from a dietitian, offering a digital nutrition platform provides a scalable yet evidence-based solution that can ultimately drive down medical costs as employees achieve weight management goals.

Nutrium Care’s observational findings provide motivation for pragmatic randomized evaluations to more directly test causal effects and durability of outcomes beyond 12 months. This need is increasingly recognized within the digital nutrition field, where planned randomized trials have begun to examine longer-term effectiveness and maintenance of digital programs [51]. Further work is also needed to characterize platform use patterns associated with optimal outcomes, for example, by identifying combinations of engagement behaviors that predict clinically meaningful weight loss and maintenance. Finally, given the scale of obesity-related costs, formal economic evaluation is an important next step. Modeling should consider (1) health care use offsets, (2) productivity impacts, (3) program delivery costs (including clinician time), and (4) subgroup targeting where marginal benefit is highest (eg, higher baseline BMI categories).

## Supplementary material

10.2196/94732 Multimedia Appendix 1 Findings from sensitivity analyses.

## Abbreviations

CEM: coarsened exact matching
DAU: days of active use
GLP-1: glucagon-like peptide-1
RD: registered dietitian
TTM: transtheoretical model
