# Supplementary material for: Weight Loss and Engagement Among Active Users of a Dietitian-Led Digital Nutrition Platform: Retrospective Cohort Study
Source: JMIR Mhealth Uhealth. 2026 Sep 15;14:e94732. doi: 10.2196/94732 (PMC13577621; doi:10.2196/94732)
Supplement: Supplementary file 1 [file mhealth-v14-e94732-s001.docx]

**Supplemental Figure 1.** Unrestricted cohort: Absolute weight loss at three and six months in kg.

**Supplemental Figure 2.** Unrestricted cohort: Threshold weight outcomes at three and six months.

**Supplemental Figure 3.** Threshold weight outcomes at three and six months among active users with the last observed weight measurement carried forward.

**Supplemental Table 1.** Unrestricted cohort: Difference in weight loss at three months by engagement metric.

| **Engagement metric (N=23,024)** | **n (%)** | **Weight loss difference, kg** | **95% CI, kg^1^** |
| --- | --- | --- | --- |
| Bi-monthly appointment | 16,902 (73%) | 1.2 | 1.0-1.3 |
| 10+ days of active use per month | 7,960 (35%) | 0.9 | 0.8-1.0 |
| Weekly food logging | 7,563 (33%) | 0.5 | 0.4-0.7 |
| Weekly water intake logging | 6,159 (27%) | 0.6 | 0.5-0.7 |
| Weekly exercise logging | 7,032 (31%) | 0.4 | 0.2-0.5 |

^1^All estimates P<.001
